# Links between Nutrition, Infectious Diseases, and Microbiota: Emerging Technologies and Opportunities for Human-Focused Research

**DOI:** 10.3390/nu12061827

**Published:** 2020-06-19

**Authors:** Manuela Cassotta, Tamara Yuliett Forbes-Hernández, Ruben Calderón Iglesias, Roberto Ruiz, Maria Elexpuru Zabaleta, Francesca Giampieri, Maurizio Battino

**Affiliations:** 1 Centre for Nutrition and Health, Universidad Europea del Atlántico (UEA), 39001 Santander, Spain; manucassotta@gmail.com (M.C.); ruben.calderon@uneatlantico.es (R.C.I.); roberto.ruiz@uneatlentico.es (R.R.); 2 Department of Analytical and Food Chemistry, Nutrition and Food Science Group, CITACA, CACTI, University of Vigo, 36310 Vigo, Spain; tforbes@uvigo.es; 3 Dipartimento di Scienze Cliniche e Molecolari, Facoltà di Medicina, Università Politecnica delle Marche, 60131 Ancona, Italy; mariaelexpuru@gmail.com; 4 Department of Clinical Sciences, Faculty of Medicine, Polytechnic University of Marche, 60131 Ancona, Italy; 5 College of Food Science and Technology, Northwest University, Xi’an 710069, China; 6 International Research Center for Food Nutrition and Safety, Jiangsu University, Zhenjiang 212013, China

**Keywords:** microbiota, infectious diseases, nutrition, human-based methods, gut-on-a-chip, gut-organoids, third-generation sequencing

## Abstract

The interaction between nutrition and human infectious diseases has always been recognized. With the emergence of molecular tools and post-genomics, high-resolution sequencing technologies, the gut microbiota has been emerging as a key moderator in the complex interplay between nutrients, human body, and infections. Much of the host–microbial and nutrition research is currently based on animals or simplistic in vitro models. Although traditional in vivo and in vitro models have helped to develop mechanistic hypotheses and assess the causality of the host–microbiota interactions, they often fail to faithfully recapitulate the complexity of the human nutrient–microbiome axis in gastrointestinal homeostasis and infections. Over the last decade, remarkable progress in tissue engineering, stem cell biology, microfluidics, sequencing technologies, and computing power has taken place, which has produced a new generation of human-focused, relevant, and predictive tools. These tools, which include patient-derived organoids, organs-on-a-chip, computational analyses, and models, together with multi-omics readouts, represent novel and exciting equipment to advance the research into microbiota, infectious diseases, and nutrition from a human-biology-based perspective. After considering some limitations of the conventional in vivo and in vitro approaches, in this review, we present the main novel available and emerging tools that are suitable for designing human-oriented research.

## 1. Introduction

The interaction between nutrition and infectious diseases has always been recognized. Before the era of antibiotics, diet was considered an essential part of the management of infections. Malnutrition, including undernutrition and overnutrition, can increase susceptibility to infectious diseases and amplify the severity of an infection, which in turn, can worsen malnutrition [1]. Thanks to the advancements in next-generation sequencing (NGS) technologies, the gut microbiota has been emerging as an integral mediator in the complex relations between food, the human body, and infectious diseases. The complex community of microorganisms inhabiting an animal’s or human’s digestive tract constitutes the gut microbiota and their collective genetic content constitutes the gut microbiome. High-throughput comparative metagenomics and meta-transcriptomics enabled by the development of NGS platforms have led to an unprecedented understanding of human, animal, and environmental microbiomes, and have shown that the gut microbiota is comparable to a virtual organ or emergent system, whose properties need to be integrated into host biology and physiology [2].

The microbiota that inhabits the human gut is crucial in regulating gut physiological functions and all body homeostasis. Such physiological functions include aiding digestion, producing metabolites from undigested fibers, regulating drug metabolism [3], impacting the development of the immune system during the early life stages [4], modulating immune responses, and protecting the host from pathogens and infections [5,6]. Changes in the gut-associated microbial community composition and diversity have been associated with several diseases [7,8,9,10,11], including infections in humans [12,13,14,15,16]. The composition of the microbiota can offer either resistance or assistance to invading pathogenic species. Differential susceptibility to *Campylobacter jejuni* infections was shown to depend on the species composition and diversity of the microbiotas in an epidemiological study of Swedish adults [17]. Several metabolites, such as short-chain fatty acids (SCFAs) that are produced by the microbiota, are important determinants of the interactions between the microbiota and pathogenic bacteria in the gut [18].

Nutrition profoundly affects the composition of the microbiota and the concentration of metabolites in the gut, which has repercussions for the physiology, immunity, and susceptibility to infectious diseases of the host [19]. The functions that the gut microbiota play in the human body can be quite easily disrupted or influenced by a wide variety of nutritional factors, including dietary habits [20], antibiotics, probiotics, and prebiotics [21]. Several nutritional studies described the impact on gut microbiota composition and the function of micro- and macro-nutrients, such as carbohydrates, novel food components, and food additives, as well as low-fat/high-fiber diets, high- or low-protein diets, diets containing high-fat/low-fiber fruits, and vegetables [20,22,23,24]. Nutrients can directly affect the gut microbiota by promoting or inhibiting microorganism growth or indirectly influencing the host’s metabolism and its immune system, or passively incorporating food-derived members into the microbiota, such as *Candida* and *Penicillium* fungi and lactic acid bacteria [25,26]. Likewise, if food is contaminated with pathogens, it can influence the development of gastrointestinal infections [27], food poisoning [28], and systemic infectious diseases [29,30].

On the other hand, infectious diseases could impact the microbiota diversity and composition by determining dysbiosis [31], which in turn, increases the susceptibility to infections.

Despite the microbiota emerging as an important modulator in the interaction between diet and infectious diseases, research findings into the role of the microbiota in the field of infectious diseases remain rudimentary.

The complexity of a holistic understanding of the cause-and-effect relationships in humans makes human studies difficult and limited, most notably because of the challenges in accessing the human gut, making disease modeling essential. Traditional 2D cell cultures or ex vivo models have been invaluable in the study of the gut microbiome and infectious diseases, but excessively simplistic cellular models, often utilizing nonhuman cells cultured under non-homeostatic and non-physiologic in vitro conditions, could have hampered, to some extent, the understanding of the complex relationships between the human host, gut microbiome, and pathogens in vivo. On the other hand, although animal models have provided extensive insights into the host–microbiota–pathogens interactions regarding diet, especially in developing mechanistic hypotheses, they fail to adequately recapitulate the complexity of human conditions and therapeutic responses.

In the last few years, there have been large steps forward in microfluidics, tissue engineering, computing power, artificial intelligence, etc., which have brought about an amazing array of tools and research approaches that are offering bold new ways to study the human gut microbiome regarding pathogens and the effect of nutrition in a human-biology-based setting. In addition, the field of metagenomics and other “omics” disciplines has greatly expanded due to improvement in sequencing technologies, namely “third-generation sequencing,” as well as analytical and single-cell techniques, allowing for a more comprehensive characterization of microbial communities and host–microbe interactions. These tools and approaches could potentially yield profuse and meaningful human-relevant data, allowing for a better understanding of human pathophysiology, while helping to reduce the number of animals employed in biomedical research, eventually replacing them. After discussing some of the major limitations associated with traditional in vivo and in vitro models, in this review, we present a list of the novel available and emerging tools and approaches used to study the interactions between the human host and the gut microbiome and pathogens through a more holistic representation of the human in vivo microenvironment. The potential opportunities regarding modifying and shaping the microbiota through nutritional interventions to treat or prevent infectious diseases will be also considered.

## 2. Limitations of Traditional In Vivo and In Vitro Models

Animal models, including mice, fish, and insects, have been extensively used to analyze host–microbiome interactions and their contributions to pathophysiology in infectious diseases regarding diet. Mice are the model of choice for most studies in this emerging field, allowing for manipulations in the gut microbiota and host to be studied in a controlled experimental setup [32]. Manipulations include host genetic background manipulation (gene knockouts); gut microbiota composition manipulation (controlled inoculation in germ-free or gnotobiotic mice, i.e., germ-free mice administered with human microbes); and ecosystem interventions, including dietary interventions, antibiotic treatment, and fecal transplantations.

Although animal models have provided critical insight into how host-microbiota homeostasis is constructed and maintained [33] or influenced by infections or dietary factors [34], they do not faithfully represent the human body and they are not suitable models to assess drug efficacy nor to evaluate complex research questions [35,36,37]. Translating results from murine models to humans remains elusive due to the existence of several key differences between the two systems [38,39], including the anatomy and function of the mouse and human intestinal tract, immune system and interaction with pathogens [40], gut microbiota composition, feeding pattern, genetic background. Besides, since crosstalk between gut microbiota and the host is host-specific, observations in mouse models might not be translatable to humans.

Although the gastrointestinal tracts in both humans and mice are composed of anatomically similar organs, the anatomy of the mouse and human intestinal tract also have prominent macroscopic and microscopic differences, which might be shaped by their diverging diets, feeding patterns, body sizes, and metabolic requirements; for example, the average ratio of the intestinal surface area/body surface area differs critically between the two species over different sections of the gut [41,42]. The intestinal tract of mice and humans also differs in histological features: the mouse colon is composed of thin muscularis mucosae with no distinct sub-mucosa, whereas the human colon is covered by a thicker mucosal wall. Another difference is the presence of transverse folds along the length of the colonic mucosa in humans, whereas these folds are limited to the cecum and proximal colon in mice. These differences in colonic micro-compartmentalization and structure might contribute to the creation of diverse ecological micro-niches presenting different microbial communities [43]. There are also several notable differences at the cellular level, e.g., the distribution of mucin-producing goblet cells and the Paneth cells. Paneth cells secrete antimicrobial compounds into the lumen of the small intestine. In mice, these cells are completely absent in the colonic mucosa and are exclusively found in the cecum, whereas they are present in the cecum and proximal colon of humans. Furthermore, mice and humans differ in the amount of defensins (peptides involved in the host defense) secreted by Paneth cells, as well as their storage and secretion [44,45,46]. These differences in the distribution and functioning of both goblet and Paneth cells between the two organisms suggest differences in local immune responses, which might shape the composition of the gut microbiota. In addition to the anatomical and histological differences, the physiology of the intestinal tract of mice and humans also differs, e.g., the overall intestinal transit time in mice is up to ten times as fast as humans. This is compatible with the total metabolic rate, which is approximately seven times higher in mice compared to humans [43]. Another factor to be considered is that mice are coprophagic. Coprophagy contributes to the nutritional value of their diet by ensuring some vitamins and fatty acids that are produced by microbiota in the cecum are not lost via defecation but re-enter the murine intestine to be absorbed [47].

Despite the known differences in various arrays of genetics, intestinal anatomy, gut physiology, enteric immune network, metabolism, dietary behavior, etc. (all of which may also correspond to microbiome differences), few independent studies have reported the features of gut microbiome configurations in different animal species [33]. An important limitation in mouse models of human microbiota studies is the difference in bacterial composition between the two species. In humans, three enterotypes can be identified, while only two can be found in mice [48,49]. Although the phylogenetic makeup of the bacterial communities in both humans and mice seems to be similar at the phylum level, at the species level, many differences are found [50] and 85% of the murine sequences represent species that have not been detected in humans [51].

Host tolerance to microbial infections varies greatly across different species [52,53] and the intestinal microbiome differentially modulates the susceptibility to infectious diseases in different species [54,55]; therefore, it remains a challenge to translate findings obtained from animal models to humans.

Humanization of the mouse microbiome is often used to address these problems. However, microbial species seem to be critically adapted to specific hosts. Human-microbiota-associated mice have low numbers of adaptive and innate intestinal immune cells and reduced antimicrobial peptide expression when compared with mice that harbor a murine microbiota. Importantly, the mouse microbiota is known to confer better protection against *Salmonella* infection than a human microbiota in mice. These data indicate a highly specific coexistence and mutual interaction between species-associated microbiota and the host immune system, and that humanized mice cannot adequately recapitulate microbiota–host interactions in humans [56].

Traditional cell-based in vitro systems have been extensively used to study intestinal barrier, host–microbiota, and pathogen interactions. Cell cultures, especially those of human origin, have been invaluable for gaining insights into bacterial adhesion/invasion, immune function, bacterial–host interaction mechanisms, and to study the protective effects of probiotics against pathogenic bacteria [57,58,59,60,61,62,63,64]. However, using cells on a 2D monolayer and/or under static non-physiologic condition (e.g., Transwell systems) could severely affect the relevance of the results. In particular, the reliability of traditional static monolayer models may be impaired by the lack of physiological stimuli, such as the biochemical signals from other cells and the extracellular matrix, the physical and structural stimuli from the three-dimensional (3D) microenvironment, and the mechanical stimuli derived from movement (e.g., peristalsis) and the physicochemical fluxes [65]. Other drawbacks of traditionally employed in vitro models are the cancerous and/or nonhuman origin of the cells [66]. Transformed colon carcinoma lines have been very popular and have led to rich insights into the host–microbe relationship and probiotic effects [60,61,62,63,67,68]. However, these transformed cell lines have many limitations, especially from a translational perspective. Many transformed cell lines have defects in innate immune signaling [69], which confound studies regarding the innate immune response to infectious diseases; for example, Caco-2 cells did not show observable innate immune responses in a conventional model of astrovirus infection [70,71].

Another limitation of the above cell models is the lack of mature cell types that can be differentiated from cell lines: the normal intestinal epithelium consists of diverse cell types, including enterocytes, goblet cells, stem cells, enteroendocrine cells, microfold (M) cells, Paneth and tuft cells, that are not accurately represented. On the other hand, cells obtained from nonhuman (animal) tissues are not species-specific, creating concerns about the translatability to humans [72,73,74,75]. Additionally, controlling the multi-species microbial communities and their growth has remained a notable technical challenge in static in vitro models [76].

The high number of therapeutic compounds that fail to translate in clinical trials [37,77,78], coupled with increasing awareness of the ethical and scientific issues surrounding the use of animal models [38,39,79,80], highlights the need and importance for models that are more physiologically relevant to the human body to personalize treatments and better predict patient outcomes.

## 3. Emerging Technologies and Opportunities for a Human-Based Research Approach

Advances in stem cell technology, microengineering, microfluidics, high-throughput third-generation sequencing techniques, computing power, machine learning (ML), and respective multidisciplinary cooperation has allowed for the development of new technologies and approaches, which were inaccessible until a few years ago. These technologies are beginning to provide more clinically relevant data and hold immense promise for studying complex regulatory networks and crosstalk between the host, gut microbiota, pathogens, and diet in a human-focused and physiologically-relevant setting. They comprise i) human-based multi-omics approaches, including (meta)genomics, (meta)transcriptomics, (meta)proteomics, metabolomics, and epigenomics, which result from global analyses of biological samples by high-throughput analytical approaches and databases; ii) computational models; iii) patient-derived cells, including induced pluripotent stem cells (iPSCs) and their differentiated derivatives, such as organoids; and iv) tissue engineering and advanced in vitro technologies (e.g., organs- or organoids-on-a-chip and microphysiological systems).

### 3.1. Multi-Omics Approaches and Computational Models

Omics disciplines include genomics, transcriptomics, proteomics, and metabolomics, which refer to the genome, transcriptome, proteome, and metabolome, respectively, of a species, population, or community. “Omics” aims at the collective characterization and quantification of pools of biological molecules that translate into the structure, function, and dynamics of an organism or population. The start of the 21st century was characterized by rapid advances in high-throughput sequencing, high-content- and single-cell technologies, mass spectrometry (MS), bioinformatics, and computational power. NGS techniques, also known as “second-generation sequencing,” which are capable of reading the code of millions of small fragments of DNA in parallel, enabled faster sequencing with increased throughput at falling costs, which allow for the assessment of genes and genomes contained within complex microbial communities. These techniques have entirely changed the perception of the human microbiome and have paved the way for the establishment of metagenomics and metatranscriptomics.

While metagenomics is the study of the genomes in a microbial community of a given sample and constitutes the first step toward studying the microbiome, metatranscriptomics involves sequencing the complete transcriptome of the microbial community and making it possible to infer the functional profile of this community under specific conditions [81]. The emergence of NGS technologies provides information about the composition and function of the entire community and the dynamics occurring between the taxa. NGS has been a source of basic biological and translational surprises, exposing a compelling range of crucial findings. Every human being appears to carry their own individual suite of microbial strains [82,83], which are acquired early in life [84,85]; differ between environments, age, and populations [86,87]; and can be altered by diet [26,88,89]. NGS has also led to the identification and characterization of metabolic and regulatory mechanisms through which hosts and microbes interact with each other to define a healthy or diseased state in the human host [90]. For example, Vázquez-Castellanos et al. [91] assessed functional modifications of HIV-associated microbiota by combining metagenomic and metatranscriptomic analyses. The transcriptionally active microbiota was shown to be well-adapted to the inflamed environment, overexpressing pathways related to resistance to oxidative stress. Furthermore, it has been demonstrated that gut inflammation is maintained by the Gram-negative nature of the HIV-associated microbiota and the under-expression of anti-inflammatory processes, such as short-chain fatty acid biosynthesis.

Meta-omics projects are largely based on short-read technologies for the taxonomic, phylogenetic, and functional evaluation of a gut bacterial community [92,93,94]. Short-read sequencing is cost-effective and accurate; however, challenges in the assembly of short reads has limited our ability to correctly assemble repeated genomic elements and place them into genomic context, failing to profile low abundance community members at the species/strains level.

Each microbial genus in the gut includes several species and strains that may harbor significant differences in their genomes and functional capacities and it has been recognized that strain-level diversity may contribute to discrepancies in genus and species associations with health and disease [95,96]. Our knowledge often relies on a genus- or species-level taxonomic assignments that, although useful, may not be sufficient for a comprehensive understanding of the complex interconnections between the gut microbiome and human health.

In recent years, new technologies that are capable of sequencing longer strands of DNA by reading single DNA molecules have advanced and become more prominent [97]. These technologies, which are also referred to as “third-generation sequencing” or “long-read sequencing,” coupled with the advancement in bioinformatics tools and single-cell sequencing methodologies, can produce genome assemblies of unprecedented quality, even for species with low abundances [94,98,99,100]. The characterization of microbial communities using short-read NGS approaches have revealed important shifts in microbiota associated with debilitating diseases, such as *Clostridium difficile* infection. However, due to limitations in sequence read length and sequence-biases, genus- and species-level classifications have been problematic. A comparison of short-read NGS and long-read, third-generation sequencing of samples from patients treated for *C. difficile* infection revealed similarities in community compositions at the phylum and family levels, but the long-read approach further allowed for species-level characterization, permitting a better understanding of the microbial ecology of this disease. Thus, as sequencing technologies continue to improve, new species-level insights can be gained in the study of complex and clinically-relevant microbial communities, as well as the relationships between gut microbiota and infectious diseases, to evaluate the effects of probiotics supplementation [101,102,103,104,105].

Metabolomics has emerged as a technique that focuses on defining the functional status of host– microbial relationships in biological specimens, providing a detailed picture of functionality and a better understanding of physiology through the identification of the metabolites produced within the gut by the host and/or the microbial community. Most microbial metabolic processes generate by-products that influence the microbiota as well as the host homeostasis. Many studies have reported that the intestinal metabolites (both from the host and microbiota) regulate pathogen infections through the use of genome-based analysis of bacteria and high-throughput metabolomics [106,107,108]. Traditionally, nuclear magnetic resonance (NMR), proton nuclear magnetic resonance (^1^H NMR) spectroscopy, and mass spectrometry (MS) have been the primary tools used in metabolomics analyses [109,110]. Advancements in these technologies, including matrix-assisted laser desorption ionization time of flight (MALDI-TOF), secondary ion mass spectrometry (SIMS), Fourier transform ion cyclotron resonance MS, in combination with the development of single-cell techniques, have improved the throughput and accuracy of metabolomics [111,112,113], which has opened up new avenues for the study of the dynamics of pathogen–microbiome–nutrients interactions and the metabolites involved in this process in a human-based setting. (Meta-)proteomics involves the high-throughput characterization of the entire constituent profile of microbial/host proteins within a biofluid or tissue sample. An important utility of metaproteomic studies is that the identification of the protein content of a sample, coupled with insight into their interactions, abundances, and modifications, gives direct information about the true functional activity of the gut microbiota. A range of different methodologies may be used for proteomic studies, including MS, NMR, microarray-based technologies, and the most recent single-cell and ultrasensitive protein analyses [114,115].

Practically, “omics” profiling has already been applied to study host–microbiota–pathogens relationships by employing a range of different human-derived sample types and experimental models (Table 1). It can be used to characterize the genome, transcriptome, proteome, or metabolome in samples from in vitro experiments, including complex gut models such as organs-on-a-chip and microphysiological systems [116].

Validation and quantification of the data and insights from “omics” technologies depend on the computational sciences and scientists being able to bridge the biosciences and bioinformatics. To date, with advances in bioinformatics, researchers have access to a variety of computational methods to analyze “omics” data [117] and the combination of different “omic” layers, leading to a multi-omic approach [118]. Integrated multi-omics analysis has already been successfully carried out for the in-depth characterization of the human microbiome’s response to a specific nutritional intervention or environmental stimuli in both healthy and diseased conditions [119,120,121]. Some “omics” and multi-omics studies on host–microbiome interactions regarding pathogens/nutrition are mentioned in Table 1.

Nutrigenomics, which integrates different omics approaches to seek and explain the existing reciprocal interactions between genes and nutrients at the molecular level, has the potential to identify genetic predictors of disease-relevant responses to diet, which has wide appeal in the context of personalized nutrition [128,129]. Nutrigenomics has already been implemented to identify cellular and molecular targets to develop a novel hypothesis regarding the functional role of nutrition and microbiota in modulating intestinal inflammatory diseases [130]. A similar approach could be possible for other diseases, including infections.

These multi-omics approaches lead to unprecedented opportunities to comprehensively and accurately characterize microbial communities and their interactions with their environments and hosts. An important goal of multi-omics data integration is to generate and validate microbiome metabolic networks/models. Though this is still challenging, promising steps forward have been made, including the generation of over 700 genome-scale metabolic reconstructions [131], the development of tools for microbiome metabolic modeling/prediction [132,133], and the generation of interspecies metabolic network databases [134].

Information deriving from “omics” techniques may also provide a basis for employing machine learning (ML). ML consists of a series of algorithms that after being “trained,” can predict outcomes and future states in specific areas of the research arena, such as shifts in the microbiome structure and function as a result of certain factors (e.g., health vs. disease status, diet, drugs, etc.) [135,136]. ML applications in multi-omics datasets were scrutinized in a series of recent reviews [137,138,139,140].

As computational models are fairly reliant on the data they are trained on or are called upon to analyze, no model, regardless of its sophistication, can generate a useful analysis from low-quality data. Since the results returned by computational models are based exclusively on the input data and represent existing knowledge, these models are valid within the same context of that knowledge and their performance will be reduced if they are not regularly updated using novel, emerging, human-relevant data. Likewise, the development and adaptation of integrated software platforms are central to the efficient and effective use of data and for predictive computational modeling [141].

### 3.2. Human Intestinal Organoids

Organoids are stem-cell-derived and self-organized 3D clusters of organ-specific cells that incorporate many of the physiologically relevant features of the in vivo tissue, including the functionality, as well as molecular and cellular heterogeneity, of the originating organ [142]. Human organoids are suitable models for studying the mechanisms of morphogenesis and are promising platforms for disease modeling and drug screening [143]. Over the past decade, researchers have developed protocols to differentiate human stem cells into multiple lineages, obtaining several organoids, including (but not limited to) pancreas [144], liver [145], stomach [146], and intestine [147].

**Figure 1:**
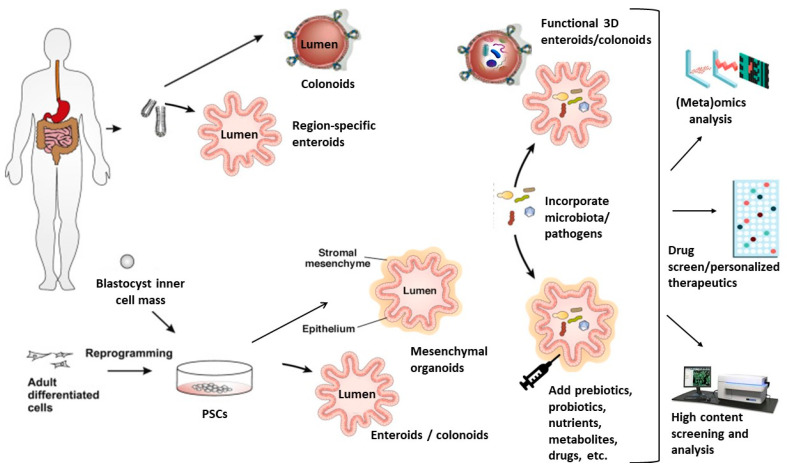
Human intestinal epithelial organoids (hiOs) generation and examples of their applications in the study of the relations between nutrition, infectious diseases, and microbiota. PSCs: Pluripotent stem cells.

Tissue-derived stem cells isolated from human intestinal biopsies or surgical specimens can be differentiated into epithelial organoids, termed “enteroids” (when derived from the small intestine) or “colonoids” (when derived from the colon) [148]. Human embryonic stem cells (hESCs) or induced pluripotent stem cells (hiPSCs) can be directed to give rise to both intestinal organoids associated with mesenchymal cell types (the so-called “mini-guts”) and epithelial organoids [147,149,150,151,152]. In this review, we use “human intestinal organoids” (hiOs) as a generic term to indicate both epithelial organoids (enteroids and colonoids) and “mini-guts” (Figure 1).

hiOs are capable of undergoing self-renewal and self-organization for an extended period and replicate many of the physiologically relevant features of the in vivo human intestinal tissue [153]. These 3D intestinal-like structures imitate the villus and crypt microarchitecture, which is a polarized epithelial layer surrounding a functional lumen, as well as the presence of all of the cell types of the intestinal epithelium, including enterocytes, goblet, tuft, Paneth, M, and enteroendocrine cells, as well as intestinal stem cells. These cells are present in proportions and a relative spatial arrangement that imitates what is observed in vivo. Since hiOs functionally mimic normal human gastrointestinal tract physiology and pathophysiology [151], they represent an effective platform to study human gastrointestinal functions and diseases [154] and are already being successfully employed to model epithelial barrier function [155,156], nutrient transport physiology during digestion [157], celiac disease [158], inflammatory bowel disease [159], and cancer [160,161,162,163]. hiOs provide unprecedented opportunities for the generation of in vitro systems with a sufficient level of complexity to model physiological and pathological diet–microbiome–host conditions [164,165] and pathogen–host interactions [72,155,166,167,168,169,170,171,172,173,174,175]. Human microbiota suspensions, pathogenic organisms, and/or nutrients can indeed be microinjected into the pseudo-lumen of organoids, which can then be recovered and assayed for microbial composition, microbial transcriptomics, metabolites, and host gene expression profiles (Figure 1). Furthermore, the addition of automated injection and harvesting systems may provide a platform for high-throughput microbiome studies [176]. hiOs have already been successfully utilized to explore host–bacterial symbiotic interactions and human viral [167,175,177,178,179], bacterial [127,155,171,180,181], and protozoan [182,183] pathogens. Human enteroids have made it possible to study host–microbe interactions that were challenging because of species-specificity that led to tolerance to infection, poor infection, and/or replication rates in animal models, including human rotavirus [167] and enterohemorrhagic *Escherichia coli* (EHEC) [171,184].

Epithelial organoids (enteroids and colonoids) have been employed to model the effects of diet and nutrients on intestinal growth and development, ion and nutrient transport, secretory and absorption functions, the intestinal barrier, and location-specific functions of the intestine [165]. hiOs responses to gut-microbiota metabolites and microbes could provide novel insights into the mechanisms by which those agents may prevent or trigger diseases, including infections, significantly extending our knowledge of diet–microbiome–host interactions [164].

I is noteworthy that human enteroids are being used successfully to study the new severe acute respiratory syndrome coronaviruses (SARS-CoV and SARS-CoV-2). Since clinical evidence suggests that the intestine may present another viral target organ, in their very recent work, Lamers et al. have demonstrated that SARS-CoV and SARS-CoV-2 can readily infect human enterocytes and that the intestinal epithelium supports SARS-CoV-2 replication. In addition, gene expression studies on enteroids have shown that interferon-stimulated genes become activated [185]. This demonstrated that hiOs serve as an experimental model for coronavirus infection and biology. Another aspect is the potential evidence on the pathophysiology of coronavirus infections modulated through the gut microbiome [186]. hiOs approaches could soon take these aspects into account by integrating, e.g., the human microbiota and/or accounting for the effects of probiotics. The treatment of a human colonoid model with the probiotic *Lactobacillus reuteri* or its antimicrobial metabolite, reuterin, before or after challenge with *S. typhimurium*, reduced the adhesion, invasion, and intracellular survival of this pathogen compared to findings for untreated cells [187]. Since the isolation of fresh crypts that contain multipotent adult stem cells is invasive and there is limited availability of live primary human cells and tissues, organoids derived from mice have been often employed [165]. However, recent advancements in culturing protocols allow for generating both epithelial-mesenchymal organoids and epithelial region-specific intestinal organoids (enteroids or colonoids) from hiPSCs [150,152,188]. hiPSC-derived organoids offer tremendous advantages if hiPSCs are harvested non-invasively and obviate the ethical dilemmas surrounding the use of hESCs. hiPSCs represent a limitless source of nontransformed patient-specific somatic cells that can be used to study the nutrient–microbiome axis in gastrointestinal homeostasis and infectious diseases in vitro in a human-relevant setting. hiOs cultures can be provided by many donors. Since they retain the genetic and biological properties of the donors, they can lead to the discovery of host-specific factors that affect susceptibility to infectious diseases and result in personalized approaches to treat individuals [173].

Stem cell 3D derived organoids overcome and surpass the limitations of the traditional 2D colonic adenocarcinoma cell lines models while providing the potential to perform mechanistic studies within a “human model” system with the same scrutiny and depth of analysis that is customary for research with nonhuman model organisms [189].

Despite the significant advances in organoid technologies and their numerous advantages over other systems, there still exist several challenges to overcome. In 3D culture, microbiological research of organoids and enteroids is very difficult. The presence of an enclosed lumen is non-physiological since secreted material from host-cells and bacteria accumulates within this central space instead of being removed by peristalsis and luminal flow. In addition, the inaccessibility of the apical cell surface makes the use of organoids experimentally challenging for transport studies, as well as exposure to living commensal microbiota or pathogens for more than approximately one day in culture. Finally, organoid cultures lack a tissue–tissue interface, mechanical forces (fluid flow and peristalsis-like movement), immune cells, and a vascular compartment, which are all crucial factors in host–microbiota homeostasis, nutrient transport, and infectious disease development.

However, there have been large steps forward in hiOs technology, including the ability to polarize the organoid cells on a 2D monolayer [172,178,190], as well as the opportunity to co-culture them with immune cell lineages, vasculature, neurons, and other cell types to make a more physiologically relevant model system [191,192]. Recent successes in the co-culture of human enteroids and macrophages/neutrophils suggest the potential for even more robust modeling of the interaction between the host and commensal or pathogenic bacteria [193]. hiOs cultured with human neutrophils imitate innate cellular responses when exposed to Shiga toxin-producing *E. coli*, including intraepithelial macrophage projections, phagocytosis, cyotokine response, loss of epithelial integrity, and the activation of stress responses that involve oxygen species [171].

In addition, progress is being made in improving the capability of organoid culture to fully mimic in vivo responses by incorporating human organoids in a millifluidic system [194] or by combining the organoid technology with a microfluidic chip system [195,196].

Although there is still significant room to improve regarding modeling the true physiology of intestinal function, hiOs have already enabled new avenues of research into host–microbial interaction and are promising tools to study the nutrients–gut microbiota–infections triangle.

### 3.3. Organs-On-a-Chip/Microphysiological Systems

Organs-on-a-chip (OoCs) are microfluidic cell culture devices in which (human) cells are cultured in engineered microenvironments that imitate the essential aspects of multicellular architectures, dynamic, tissue-tissue interfaces, physicochemical microenvironments, flow, and gradients found in the human body [197]. A wide range of tissues and organs have been modeled, including heart [198], kidney [199], brain [200], liver [201], blood vessels [202], lymphoid follicle [203], and intestine [204]. For this review, we discuss the use of OoCs to study intestinal dynamics with a specific focus on the gut microbiome and infectious diseases. OoCs models of the human intestine have been developed and successfully employed to study intestinal physiology and pathophysiology. Over the past few years, human intestine-on-a-chip models have been engineered with increasing complexity that also include neighboring channels lined by human microvascular endothelium, commensal microbiota, pathogenic bacteria, immune cells, and some even allow for the application of cyclic mechanical forces that mimic peristalsis-like deformations experienced by the intestine in vivo [205]. Peristalsis-like mechanical forces induce epithelial cells to spontaneously form polarized 3D villus-like structures that contain all the specialized differentiated intestinal epithelial cells (including adsorptive enterocytes, mucus-producing goblet, Paneth, and enteroendocrine cells) [195,206,207,208]. The resulting epithelial layer shows the basic functional properties, such as mucus production, high barrier resistance, activity of the brush border, drug-metabolizing enzymes, and high efficiency in nutrient uptake. These features allow for studies focusing on nutrient uptake and digestion, barrier function, and drug metabolism [195,206,207,208,209], as well as for co-cultures with human commensal bacteria for extended periods (up to weeks) [206,210]. Human intestine-on-a-chip or human gut-on-a-chip (hGoC) have already been used to model invasion or infection by pathogenic entero-invasive *E. coli* strains into the commensal bacterial biofilm or the host intestinal endothelium [210,211], as well as to model *Staphylococcus aureus*, *Pseudomonas aeruginosa*, and *Salmonella typhimurium* infections [212,213]. Grassart et al. [214] have successfully modeled the impact of flow and peristalsis on the human pathogen *Shigella* within a 3D colonic epithelium using hGoC technology. The authors observed that *Shigella* invasion accurately imitates what has been previously reported from clinical data. *Shigella* was also shown to leverage the intestinal microenvironment by taking advantage of the microarchitecture and mechanical forces to efficiently invade the intestine. This study is the proof-of-concept that we can use hGoCs to gain insights into infection mechanisms of human-restricted pathogens and that such models could provide a viable alternative to animals, particularly where species differences can preclude accurate extrapolation to humans. In addition, some lactic acid bacteria have been used to model the presence or biochemical contribution of probiotics in human microbiota using hGoC models [206,212,215]. Notably, it has been shown that the probiotic *Lactobacillus rhamnosus* GG improved intestinal barrier function when co-cultured in the lumen of the intestinal epithelial channel of the hGoC [206], and that the VSL#3^®^ probiotic formulation suppressed villus blunting and the loss of barrier function induced by infection with pathogenic *E. coli* in this model [210]. Thus, the hGoC approach may also be useful for the discovery of new microbiome-based therapeutics, such as genetically engineered commensal bacteria [216]. Furthermore, by integrating circulating and organ-specific human immune cells into hGoCs, they might be useful for the in vitro development of new mucosal vaccines [217]. Villenave et al. [218] demonstrated that human *Enterovirus* infection, replication, and infectious virus production can be analyzed in vitro in a hGoC microfluidic device. Since the analysis of *Enterovirus* infection is difficult in animals because they express different virus receptors than humans, hGoCs may provide suitable in vitro models for enteric virus infection and for investigating the mechanisms of enteroviruses’ pathogenesis.

**Figure 2:**
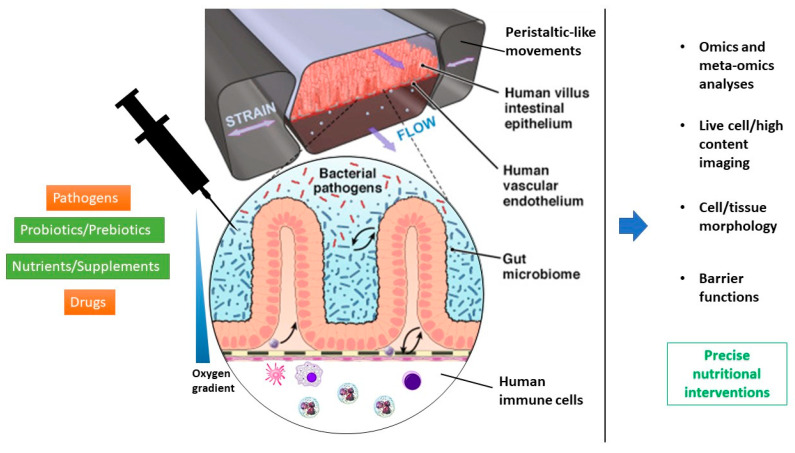
Scheme of a human gut-on-a-chip and its potential applications in the study of the interaction between the microbiome, infectious diseases, and nutrition.

OoC technology integrated with multi-omics approaches has allowed for the identification of specific human microbiome metabolites modulating enterohemorrhagic *Escherichia coli* (EHEC) pathogenesis. It has been shown that epithelial injury was greater when exposed to metabolites derived from the human gut microbiome compared to those derived from mice. The active human microbiome metabolites preferentially induced the expression of flagellin, a bacterial protein associated with the motility of EHEC and increased epithelial damage. The authors concluded that the decreased tolerance to infection observed in humans versus other species might be due in part to the presence of compounds produced by the human intestinal microbiome that actively promote bacterial pathogenicity [116]. A noticeable advantage of microfluidic OoC is the ability to integrate analytical biosensors into the culture system, thus combining living cells and sensors for the non-invasive detection of cellular physiological parameters, including O_2_, pH, protein and metabolite secretion, and cell layer barrier and cell–cell interaction, via fluorescence and confocal microscopy [219]. Jalili-Firoozinezhad and colleagues set out to develop an experimental intestine-on-a-chip system that permits the control and real-time assessment of physiologically relevant oxygen gradients and can support dynamic interactions between living, mucus-producing human intestinal epithelial cells and a complex community of living human aerobic and anaerobic commensal gut microbes. When compared to traditional aerobic coculture conditions, the establishment of a transluminal hypoxia gradient in the chip increased intestinal barrier function and sustained a physiologically relevant level of microbial diversity, containing over 200 unique active taxonomic units from 11 diverse genera and an abundance of obligate anaerobic bacteria, with ratios of Firmicutes and Bacteroidetes similar to those observed in human intestines [220]. This model may serve as a human-relevant in vitro discovery tool for the development of microbiome-related therapeutics, probiotics, and nutraceuticals, as well as for examining the biological interactions of food products, the microbiome, and infectious diseases. Figure 2 shows a schematic representation of a hGoC.

Integrating patient-specific or hiPSC-derived intestinal 3D cell constructs, local immune cells, pathogens, and the commensal microbiota into OoCs might create a highly defined and controllable translation platform that should accelerate the discovery of new drugs and/or personalized precision probiotic therapeutics. Maurer et al. have established a 3D immunocompetent intestine-on-a-chip model as an in vitro platform for functional and microbial interaction studies [221]. This model allows for a detailed characterization of the immune response, microbial pathogenicity mechanisms, and quantification of cellular dysfunction attributed to alterations in the microbial composition. The authors examined the microbial interaction between probiotic *Lactobacillus rhamnosus* and the opportunistic pathogen *Candida albicans*, showing that pre-colonization of the intestinal lumen of the model by *L. rhamnosus* reduces *C.*-*albicans*-induced tissue damage, lowers its translocation, and limits the fungal burden. Utilizing a similar model, Graf et al. established an in vitro system that can be used to experimentally dissect the commensal-like interactions of *C. albicans* with a bacterial microbiota and the host epithelial barrier, discovering fungal shedding as a novel mechanism by which probiotics contribute to the protection of epithelial surfaces [5]. While an OoC construct is designed to imitate the structure and function of a single human organ or organ region, microphysiological systems (MPSs) consist of interacting OoCs or tissue-engineered, 3D organ constructs that use human cells. OoCs coupled together to create a MPS provide the ability to analyze multiorgan interactions and offer the opportunity of providing an unprecedented physiological accuracy for disease modeling and drug discovery in vitro, allowing for investigating the complex physiological and pathophysiological responses of cells and tissues at a multi-organ level [222]. MPSs have been developed for several organ systems, including liver–skin–intestine–kidney [223], gut–microbiome–brain [224], and gut–liver [225]. The interaction between the liver and the gut microbiota, with their reciprocal influence on biosynthesis pathways and the integrity of the intestinal epithelial barrier, has been documented. Dysbiosis or liver disorders lead to intestinal epithelial barrier dysfunction, altering membrane permeability [226]. The potential of MPSs to accurately model drug uptake and metabolism in the human body has recently been shown in the context of metabolites produced by the microbiota. Vernetti et al. [227] have demonstrated that an intestine–liver–kidney-on-a-chip system coupled with an intact blood–brain barrier/neurovascular unit adequately modeled in vivo trimethylamine (a by-product of the microbiome) metabolism. Specifically, trimethylamine that was microinjected into the intestinal compartment was found in the basolateral media and was subsequently metabolized to trimethylamine *N*-oxide by the liver module and then secreted into the lumen of the kidney module, which occurs in vivo. The study also revealed a novel finding that trimethylamine *N*-oxide crosses the blood–brain barrier, highlighting the potential of such systems to improve our understanding of human pathophysiology.

Integrating gut–liver organ-on-a-chip systems with pathobionts/pathogens and immune cells might allow for future study into the interaction between microbiota, pathogens, and the effect of nutrients in a more complex multi-organ context.

## 4. Discussion

While traditional animal and cell culture models of gut microbiota and infectious diseases have been useful for elucidating some of the mechanisms underlying microbiota and nutrition relationships, the use of non-human (animal) models to mimic the complex interplay between host–microbiota–pathogens and the effects of nutritional pro- and prebiotic interventions may potentially be misleading in light of the numerous interspecies differences. In addition, although some problems of external validity can be overcome by improving the animal models, the problem of species differences can never be overcome and will always undermine external validity and the reliable translation of preclinical findings to humans, emphasizing the need to focus on human-relevant research methods and technologies [37,228]

Here we described some new technologies, tools, and approaches that could be employed in an integrated human-focused framework that is suitable for investigating the interaction between gut microbiota, nutrients, and infectious diseases. To our knowledge, this is the first review discussing the applicability of human-based methods and models to microbiome research related to infectious diseases and nutrition.

In recent years, the shift toward a new human-biology-focused paradigm has been broadly encouraged in toxicology and regulatory testing [229], but also in other research fields, including immunology, human infectious diseases, and nutritional research [7,80,230,231,232,233,234,235,236,237,238,239,240,241].

The envisioned human-based framework will not only increase the human relevance and translatability but will also contribute to the reduction and/or replacement of animals conventionally used in microbiota and infectious diseases research, which is very important considering the rising concerns for the ethical justifications of the use of animals in research. The EU legislation governing animal experimentation (European Directive 2010/63/EU on the protection of animals used for scientific purposes) [242], as well as the U.S. regulatory and research agencies in both environmental and medical arenas [243], actively support animal replacement/reduction in accordance with the 3R principle (animal replacement, reduction, refinement).

Our ability to generate 3D engineered tissue models from human embryonic stem cells or induced pluripotent stem cells continues to make rapid progress. We may soon be able to assemble them into a miniature gastrointestinal system in a dish or even on a chip [244]. This would facilitate the manipulation and analysis of digestion in a reproducible, accurate, precise, and large-scale manner, and allow for exploration of the individual genetic and microbial diversity regarding pathogens and the effects of probiotics or nutrients.

The microbiota responds dynamically to nutritional cues, it changes with age, and has close interactions with its host through the immune system and metabolites. It is also extremely variable between individuals and societies, which represents the main source of metabolic unpredictability [245]. Although the in vitro modeling of the gut microbiome is still in the early stages, and some bacterial strains are not easily cultured in the lab, the ever-expanding fine-scale knowledge of microbiota functions and innovative culture systems is very promising [100,204,220]. Experimenting with this system will teach us about human–microbiota interactions and explore how metabolic variability relates to microbiome composition.

Since the stomach, duodenum, jejunum, ileum, and colon have different digestive functions and encounter food at different digestive stages, in vitro systems should be designed such that stem-cell-derived intestinal segments form separate chambers that pass food and chyme at diverse stages of digestion through the “lumen” of the system. Tailored culture conditions and chip mechanics will be essential to support the introduction of food and provide appropriate conditions for gut microbiota to flourish [244]. The various parameters could be well controlled to allow for collecting high quality, reproducible data. For example, it will be possible to sample fluids containing absorbed metabolites from different positions in the in vitro model and understand how they affect or are affected by gut microbiota, pathogens, probiotics, a sudden change in diet, antibiotics, or fasting and feeding. “Biopsies” or fluid samples from the in vitro system could teach us about the proteomic and transcriptomic profile in response to different cues, with a high temporal resolution. The ability to genetically modify stem cells that are employed to make the tissues and organs will be extremely valuable in defining which genes function in which cells to affect the phenotype. Advances in gene editing using TALEN (Transcription Activator-like Effector Nucleases) and CRISPR (Clustered Regularly Interspaced Short Palindromic Repeats) technologies allow for testing both the loss and gain of function for specific genes and tissues [246]. Building reliable in vitro models capable of mimicking digestion and human gastrointestinal physiopathology is a monumental task demanding the collaborative efforts of labs from several fields in biology, chemistry, and engineering [247]. Some crucial parts of the system are missing and incorporation of the stem-cell-derived tissues in a bioengineered system will be challenging. However, considering the advancement in the last decade in stem cell and bioengineering arenas, there is good reason to believe that creating a human-biology-based and human-relevant model of gastrointestinal tract suitable for nutritional studies is possible. Moreover, it is important to notice that these devices do not attempt to recreate the highly complex composition and dynamics of the human gastrointestinal tract and the gut microbiota; rather, they aim to recapitulate key features of human physiology.

It has to be said that in the proposed strategic framework, the use of patient-derived cellular models, such as intestinal organoids or gut-on-a-chip, and the application of (meta)omics readouts while aiming for human relevance would still represent the lower level/scale of (“wet”) lab research. Consequently, wide-scale computational approaches, together with large-scale epidemiological data sets represent the crucial tools required to produce a higher level/scale and to establish systemic correlations between signaling pathways, (meta)-omics perturbations, humans heterogeneity, and the effect of diet and/or nutritional interventions on infectious diseases development and outcomes. The strategy should involve large, adequately powered international studies that recruit patients and controls to collect clinical data, detailed dietary assessments, host genetics, immune phenotyping, and multi-omic gut-microbiome markers. The international approach would enable the inclusion of populations from different regions with different backgrounds, various dietary patterns, and environmental exposures. This broad and collaborative approach is essential for unraveling the determinants of clinical outcomes of infectious diseases and for designing targeted therapeutic and preventative measures. The effects of high fiber, freshly fermented, and diverse foods or probiotics should also be examined as preventative and mitigating measures.

**Figure 3:**
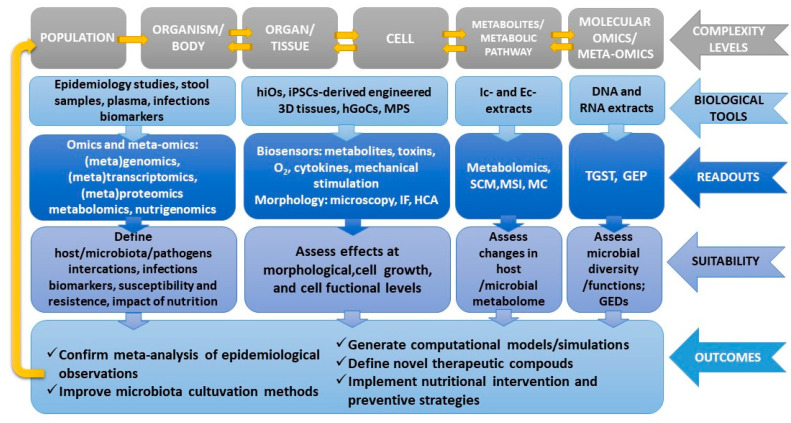
Overview of the novel available tools and readouts applicable to study the links between nutrition, infectious diseases, and microbiota in a human-relevant setting that accounts for multiple levels of complexity, from the molecular to the population level. MPS, microphysiological systems; iPSCs, induced pluripotent stem cells; IF, immunofluorescence; HCA, high-content analysis; MS, mass spectrometry; GEP, gene expression profiling; Ic and Ec, intracellular and extracellular; GEDs, gene expression dysregulations; MC, mass cytometry; MSI, high-resolution mass spectrometry imaging; SCM, Single-Cell Metabolomics; TGST, third-generation sequencing technologies; hiOs, human intestinal organoids; hGoCs, human gut-on-a-chip.

The feasibility of the envisioned human-based strategy necessarily requires the establishment of an integrated, collaborative strategy to investigate the relationship between nutrition, microbiota, and infectious diseases at multiple levels of complexity (from gene expression to protein, cells, and tissues/organs at the individual and population levels) (Figure 3).

## 5. Conclusions

Advanced human-relevant 3D cellular models, high-throughput (“omics” and meta-omics) readouts, and computational models, together with data obtained from the meta-analysis of epidemiological and interventional studies, are among the ideal tools used to investigate the complex relations between nutrition, microbiota, and infectious diseases in a human-biology-based milieu, as well as to develop new microbiome-related therapeutics or to implement personalized nutritional interventions.

Although most of the methodologies and approaches described in this review are still in their infancy, they are already yielding meaningful human-relevant data. In our opinion, the development and application of these approaches should be encouraged, while funding for research focusing on human-centered models, rather than “improved” animal models, should be prioritized.

## Figures and Tables

**Table 1:** Examples of the applications of human-based (meta)omics and multi-omics approaches to investigate host–microbiota–pathogen–nutrition relationships.

Type of Omic Approach	Type of Model	Description/Major Findings	References
Multi-omics	hGoC	Identification of specific human microbiome metabolites modulating EHEC pathogenesis	[116]
Metagenomics/Metabolomics	HITChip/M-SHIME	In-depth microbial characterization of luminal and mucosal gut microbes	[122]
Metagenomics/Metabolomics	Human subjects/stool samples	Characterization of the gut microbiome of individuals living in the Amazon showed striking differences in the microbial communities from these two types of populations	[123]
Metabolomics	In vitro SIHUMIx	Analysis of the impact of functional food on the microbic metabolic pathways	[124]
Multi-omics	Human subjects/stool and plasma samples	Investigation of the interplay between the human gut microbiome and the host metabolism	[125]
Meta-proteomics	Human subjects/stool samples	Extensive microbiome comparison between infants and the identification of previously undetected microbial functional categories	[126]
Transcriptomics/Metatranscriptomics	hiOs	Exploration of the interaction of *Salmonella enterica* serovar *Typhimurium* with hiOs; clear changes in transcriptional signatures were detected	[127]

Abbreviations: hGoC, human gut-on-a-chip; EHEC, enterohemorrhagic *Escherichia coli;* HITChip, human intestinal tract chip; M-SHIME, mucosal-simulator of a human intestinal microbial ecosystem; SIHUMIx, simplified intestinal human microbiota; HiOs, human intestinal organoids.
